# Calculation model for ventilation friction resistance coefficient by surrounding rock roughness distribution characteristics of mine tunnel

**DOI:** 10.1038/s41598-022-07115-5

**Published:** 2022-02-24

**Authors:** Ke Gao, Zhipeng Qi, Yujiao Liu, Jinyi Zhang

**Affiliations:** 1 College of Safety Science and Engineering, Liaoning Technical University, Huludao, 125105 Liaoning China; 2 Key Laboratory of Mine Thermodynamic disasters and Control of Ministry of Education, Ministry of Education, Huludao, 125105 Liaoning China

**Keywords:** Fluid dynamics, Coarse-grained models

## Abstract

Real-time mine ventilation network solution is the core way to realize the actual intelligent ventilation, and ventilation friction resistance coefficient is a significant parameter of network solution. With the help of fractal theory to characterize the three-dimensional roughness characteristics of tunnel surrounding rock. A method to describe the roughness by fractal dimension and fractal intercept. We put the fractal dimension and fractal intercept into Matlab to randomly generate three-dimensional laser scanning data of tunnels. The fusion of the two fractal parameters made the three-dimensional roughness surface information more comprehensive. It has been applied to field practice accurately. Compared to the simulation results show that the relative error of the new prediction results is 3%. Comprehensive evaluation analysis shows that the new friction wind resistance formula can fully reflect the influence of three-dimensional rough surfaces on airflow friction resistance. With the help of three-dimensional laser scanning technology, we can calculate the airflow friction resistance of the tunnel quickly and accurately, which provides a reference for the development of key technology and the theory of intelligent ventilation parameter measurement.

## Introduction

Mining ventilation technology has become an irreversible trend of information-based and agent development. Realize real-time control of ventilation network, the coefficient of friction resistance must be obtained^1^.The coefficient of friction resistance is directly related to the characteristics of surrounding rock. The investigation of the surrounding rock of roadways has also obtained fruitful achievement^2–4^. Studies show that the frictional wind resistance of complete turbulence is only related to inherent properties such as tunnel length, cross-section area, perimeter, and relative roughness^5^. It is difficult to measure relative roughness directly. At present, the ventilation friction resistance coefficient can be obtained by querying the value from the same type of tunnel, or by inverse calculation of airflow volume, airflow velocity, and pressure by adjustment method^6^. For the former, we can refer to the exact roughness or friction coefficient values in the manual, and the results often lead to worse errors than the actual values. The latter requires tedious work, and some mines have hundreds of tunnels. As the mining depth and the number of working places increases, some mines even have thousands of tunnels. Neither of the two methods can satisfy the real-time calculation of an intelligent ventilation network. Developing a new way to obtain friction wind resistance has become a research hotspot.

Three-dimensional point cloud modeling has gone for buildings widely, roads, and Bridges due to its convenience and strong adaptability^7–9^, but it is seldom applied in roadway research. It only proposed a method to obtain the wall roughness based on point cloud data and calculate the airflow friction resistance of the tunnel^10,11^, which was too simple and more like a procedural operation method. Many friction solutions are also based on one-dimensional relative roughness, which loses roughness information on tunnel length and cross-section that is not the same with three-dimensional rough surface characterization. Therefore, it is necessary to establish a new method for calculating airflow friction resistance that can characterize the three-dimensional rough surface of the tunnel.

Fractal geometry provides a method for the characterization of three-dimensional irregular geometric surfaces and can well avoid the problem of excessive roughness parameters in statistical models^12–15^. Therefore, the fractal theory is used in three-dimensional roughness characterization widely. Many kinds of pieces of literature have discussed the fractal parameters of rock and soil pores, cracks, and rock joints^16–18^. In some works of literature, the roughness is characterized by the second-order fractal dimension and the large convex and the second-order convex^19^. We put forward a fractal roughness evaluation system to influence the anisotropy and measurement scale of wall rock mass surface^20,21^. To apply fractal dimension to empirical formulas in various fields, some scholars studied the relationship between fractal dimension and three-dimensional roughness parameters to establish the nonlinear law between them^22–24^.

In conclusion, the development of 3D scanning modeling provides a way to propose new roughness algorithms to obtain digital information of wall morphology. The wide application of fractal theory in the geotechnical field provides a new means for roughness characterization based on point cloud data. This study proposes a new prediction algorithm for ventilation friction resistance coefficient with three-dimensional point cloud and fractal theory. It not only contains the innovation of one-dimensional roughness parameter characterization in the traditional prediction formula but also variations in length and cross-section are incorporated to make the physical meaning more explicit. A more effective calculation method of airflow friction resistance which can represent the three-dimensional roughness of surrounding rock is deduced based on fractal theory. Realize the intelligent acquisition method of tunnel friction and resistance based on 3D laser scanning technology. It avoids the subjectivity of reference value and the complexity of inverse calculation by the difference method. It put forward a reference to the development of friction resistance theory and key technology of intelligent ventilation parameter measurement.

## Fractal roughness for surrounding rock

**Figure 1:**
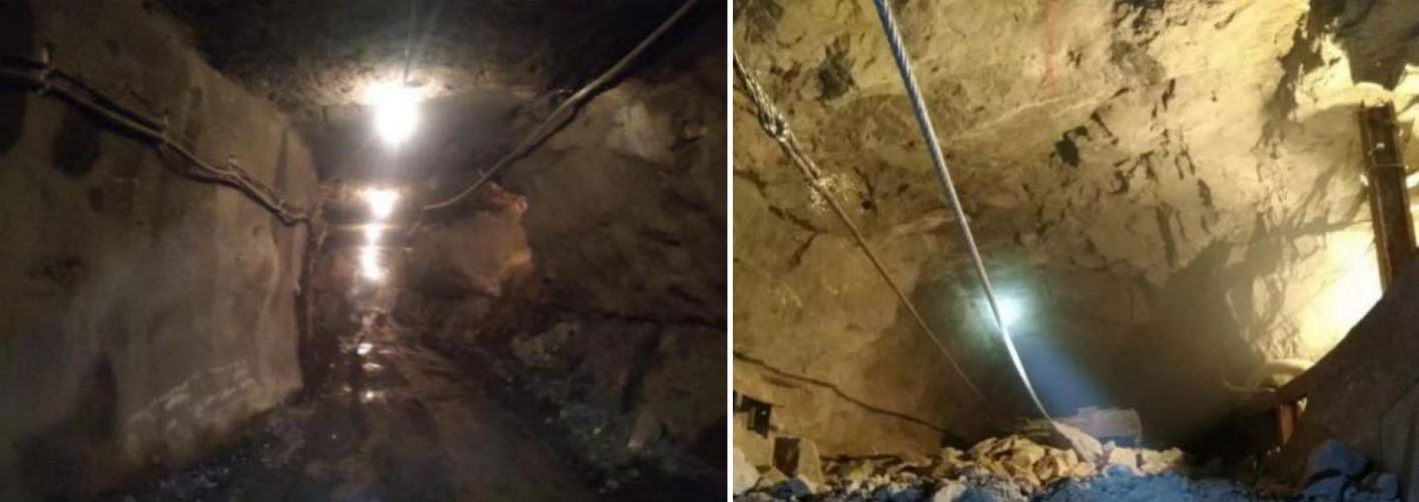
Rough surface.

Figure 1 shows the rough surface after excavation. The flow resistance generated by the rock surface mainly depends on the morphology of the rock surface. A description of the rock surface roughness characteristics is the basis for the friction resistance.

**Figure 2:**
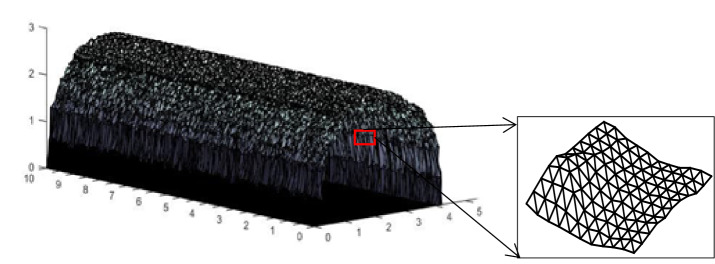
Tunnel model diagram.

We obtained the point cloud coordinates of the rock surface by the 3D scanner to study the relationship between the surface morphology and flow friction in theory. All the point clouds are traversed, and the adjacent point clouds are used to form a triangular mesh to cover the surface. Figure 2 shows the rough surface model.

**Figure 3:**
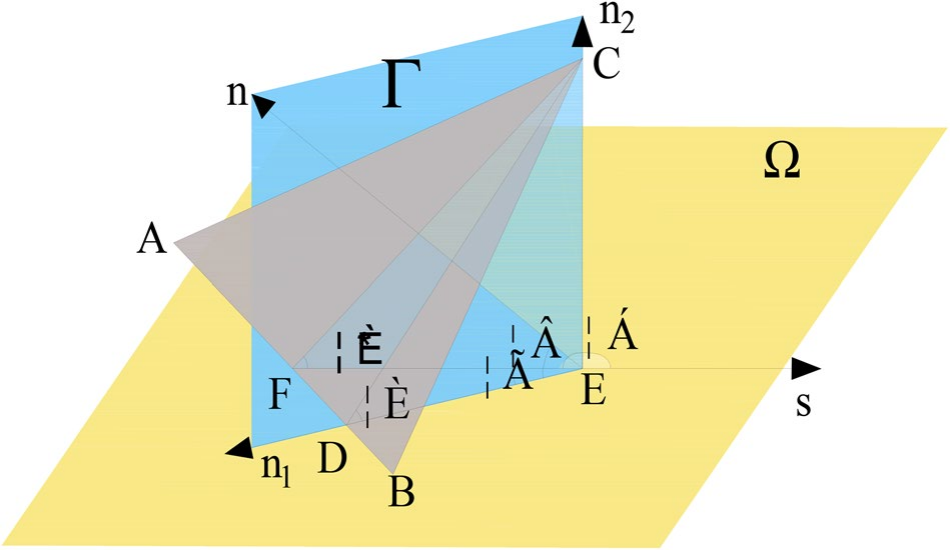
Calculation diagram.

Furthermore, focusing on the mesh wind flows along with a specific heading to a triangular network created in the graph, the contributions of the unit of the flow obstruction capability might be expressed in Fig. 3. $\Omega$ is flow level; $\Gamma$ is the triangular element level; *n* is the normal vector of a triangular element, where $n = AB \times AC$ is the flow direction vector; and ***n***_**1**_ is a part from *n* in the flow level, where $n_{1} { = }\left( {a,0,c} \right)$. ***n***_**2**_ is a part from *n* that is perpendicular to the flow level; $\theta$ is inclination angle; $\alpha$ is an angle between wind direction and inclination; $\theta^{*}$ is obstacle angle (along wind direction).

The following formula can calculate the tangent value of the obstacle angle of the triangular grid. This angle represents the ability of meshes to hinder flow. The larger the obstacle angle is, the stronger the flow friction is

1 $$I_{i} = \tan \theta^{*} = - \tan \theta \cdot \cos \alpha ,$$

2 $$\sin \theta = \cos \left\langle {{\text{n}},\;{\text{n}}_{1} } \right\rangle ,$$

3 $$\cos \alpha = \cos \left( \frac{{{\text{n}}_{1} \cdot {\text{s}}}}{{\left| {{\text{n}}_{1} } \right|\left| {\text{s}} \right|}}\right).$$

Further investigation demonstrates that the blocking angle of the triangular mesh is similar to the slope angle of the concave-convex height line of the rough curve^25^. The difference is that the algorithm considers airflow direction and the geometric contour information in detail on the obstacle angle. The apparent inclination angle of the element can reflect the mechanical effect of the rock surface on the airflow more comprehensively than considering only the influence of the contour line. The above analysis only discusses whether a triangular mesh affects the airflow. Figure 2 shows that the actual surrounding rock surface is composed of multiple meshes, so it is necessary to consider the joint effect of all triangular meshes. According to the root mean square slope of the contour line, the total obstacle angle parameters are defined by a statistical method.

4 $$\theta_{{{\text{total}}}} = \arctan \left[ {\left( {\frac{1}{n}\sum\limits_{i = 1}^{n} {\left( {I_{i} } \right)^{2} } } \right)^{1/2} } \right].$$

**Figure 4:**
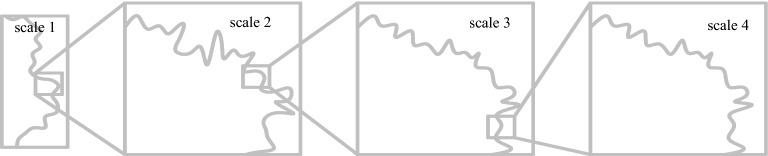
Multi-scale points density.

The tunnel surface is scanned at different scales to acquire points, and then we can use the small scale to obtain dense sections. The scanning order can be regarded as the orderly arrangement of different sections depicted by the point cloud along the tunnel. As shown in Fig. 4, *O*_*i*_ is the number of scanning sections. When the section spacing is $\Delta x$, the scanning scale is the smallest, and the points are the densest. The point density decreases as the scanning scale increases to $2 \cdot \Delta x$, $4 \cdot \Delta x$, $8 \cdot \Delta x$, and $16 \cdot \Delta x$. The largest scanning scale and the sensitivity points with the scanning scale are $16 \cdot \Delta x$, as shown in Fig. 4. The increases in scan scale do not have to be limited to integers, and the scale can present any multiples. Scanning section density changes are the spatial form of the triangular mesh changes, and the airflow obstacle angle also changes.

The unique representation of roughness can be achieved by analyzing the relationship between the density change of the point cloud in the spatial sequence and the obstacle angle. The numerical shift in the total obstacle angle $\theta_{total}$ at scale $\sigma^{2}$ is a discrete distribution. When the displacement $\Delta t$ changes slightly, the variance obeys the power–law relation with one dimension. The spatial sequence with fractal characteristics can satisfy the structure–function:

5 $${\text{var}}\left[ {{\text{z}}\left( {t + \Delta t} \right) - {\text{z}}\left( t \right)} \right]^{2} \propto C \cdot t^{4 - 2 \cdot D},$$

where var(·)—point variance; C—the constant factor of the structural function; and *D*—the structure function index. If $\Delta t{ = }t_{i + 1} - t_{i}$ and $\Delta \theta_{\Delta t} = \theta \left( {t_{i + 1} } \right) - \theta \left( {t_{i} } \right)$, the index *D* of the structure function can be calculated as follows:

6 $$\left( {{4 - 2} \cdot D} \right){\text{log}}_{10} \left( {\Delta t} \right) = {\text{log}}_{10} \left[ {{\text{var}}\left( {\Delta \theta_{{\Delta {\text{t}}}} } \right)} \right] + C,$$

where, *C* is a constant. When $\Delta t$ is small, ${\text{log}}\left( {\Delta {\text{t}}} \right)$ and ${\text{log}}\left[ {{\text{var}}\left( {\Delta \theta_{{\Delta {\text{t}}}} } \right)} \right]$ are linearly distributed in the rectangular coordinate system. The slope of ${\text{log}}_{{{10}}} \left( {\Delta {\text{t}}} \right)$–${\text{log}}_{{{10}}} \left[ {{\text{var}}\left( {\Delta \theta_{{\Delta {\text{t}}}} } \right)} \right]$ in logarithmic coordinates is *W*:

7 $$W = 4 - 2 \cdot D = \mathop {{\text{lim}}}\limits_{{\Delta {\text{t}} \to 0}} \frac{{{\text{log}}_{10} \left[ {{\text{var}}\left( {\Delta \theta_{{\Delta {\text{t}}}} } \right)} \right]}}{{{\text{log}}_{10} \left( {\Delta {\text{t}}} \right)}}.$$

The structural function index *D* is a fractal dimension, which can be expressed as:

8 $$D = \frac{4 - W}{2}.$$

The surface roughness cannot obey the power function accurately, and the point cloud cannot achieve $\Delta t$ close to zero, so formula (5) is not accurate. A new algorithm needs to be developed to obtain the structure–function index *D*. Choosing a set of spatial variations $\left\{ {\Delta {\text{t}}_{1} ,\Delta {\text{t}}_{2} , \ldots ,\Delta {\text{t}}_{{\text{k}}} } \right\}$ as the output, we can acquire the corresponding output $\left\{ {\Delta \theta \left( {\Delta {\text{t}}_{1} } \right),\Delta \theta \left( {\Delta {\text{t}}_{2} } \right), \ldots \Delta \theta \left( {\Delta {\text{t}}_{k} } \right)} \right\}$. Linear regression is used to fit these data points in double logarithmic coordinates, and the slope of the fitting line is used to replace the slope mentioned above. The higher the correlation between the fitting line and the base points is, the higher the approximation between the modified algorithm and the power law is. The fitting line slope *W* can be expressed in the following formula.

9 $$\left\{ \begin{array}{*{20}l} X_{{\text{k}}} = {\text{log}}_{10} \left( {\Delta {\text{t}}_{{\text{k}}} } \right) \hfill \\ Y_{{\text{k}}} = {\text{log}}_{10} \left[ {{\text{var}}\left( {\Delta \theta_{{\Delta {\text{t}}_{{\text{k}}} }} } \right)} \right] \hfill \\ \end{array}, \right.$$

where, *k* belongs to[*A,B*]. If the power rate relationship in the number set is established, the fitting line slope is:

10 $${\text{W}} = \frac{{\left( {B - A} \right)\sum\limits_{{{\text{k}} = A}}^{{\text{B}}} {X_{{\text{k}}} Y_{{\text{k}}} - \sum\limits_{{{\text{k}} = {\text{A}}}}^{{\text{B}}} {X_{{\text{k}}} } \cdot \sum\limits_{{{\text{k}} = {\text{A}}}}^{{\text{B}}} {Y_{{\text{k}}} } } }}{{\left( {\text{B}} - {\text{A}} \right)\sum\limits_{{{\text{k}} = {\text{A}}}}^{B} {X_{{\text{k}}}^{2} { - }\left( {\sum\limits_{{{\text{k}} = A}}^{B} {X_{{\text{k}}} } } \right)^{2} } }}.$$

In addition, the intercept of the fitting line on the y-axis is the structural function constant *C*. We can obtain two fractal parameters: fractal dimension *D* and fractal intercept *H*. Further analysis shows that the fractal dimension is a relative roughness index that measures the complexity of the surface and has nothing to do with the measurement scale. The fractal intercept is an absolute index to measure the roughness surface, which measures the overall surface fluctuation and is related to the measurement scale. It can be seen that a single fractal parameter is not comprehensive to describe the roughness of the joint surface, so it is necessary to consider the influence of fractal dimension and fractal intercept together. Therefore, two feature parameters are coupled as a parameter to describe the surrounding rock roughness, which is beneficial to improve the recognition of the surface. The fractal roughness $\varepsilon_{f}$ coupled with the weight factor can be expressed as:

11 $$\varepsilon_{f} = \sqrt {\left( {\omega_{1} \cdot D} \right)^{2} + \left( {\omega_{2} \cdot H} \right)^{2} },$$

where $\omega_{1}$ is the fractal dimension weight and $\omega_{2}$ is the fractal intercept weight. The relative weights are determined by combining the Delphi and AHP methods.

12 $$\omega _{{\text{i}}} = \left\{ {\begin{array}{*{20}l} {1,} \hfill & {{\text{i}} = 1} \hfill \\ {\frac{1}{2} + \frac{{\sqrt { - 2{\text{ln}}\left( {\frac{{2\left( {{\text{i }} - {\text{ }}1} \right)}}{{\text{n}}}} \right)} }}{6},} \hfill & {1 < {\text{i}} \le \frac{{{\text{n}} + 1}}{2}} \hfill \\ {\frac{1}{2} - \frac{{\sqrt { - 2{\text{ln}}\left( {2 - \frac{{2\left( {{\text{i }} - {\text{ }}1} \right)}}{{\text{n}}}} \right)} }}{6},} \hfill & {\frac{{{\text{n}} + 1}}{2} < {\text{i}} \le {\text{n}}} \hfill \\ \end{array} } \right.,$$

where *n* is the number of indicators; the description roughness is the fractal dimension and fractal intercept, so *n* = 2; and *i* is the importance level. If the index is equally important, *i* is the same value.

## Ventilation resistance coefficient

**Figure 5:**
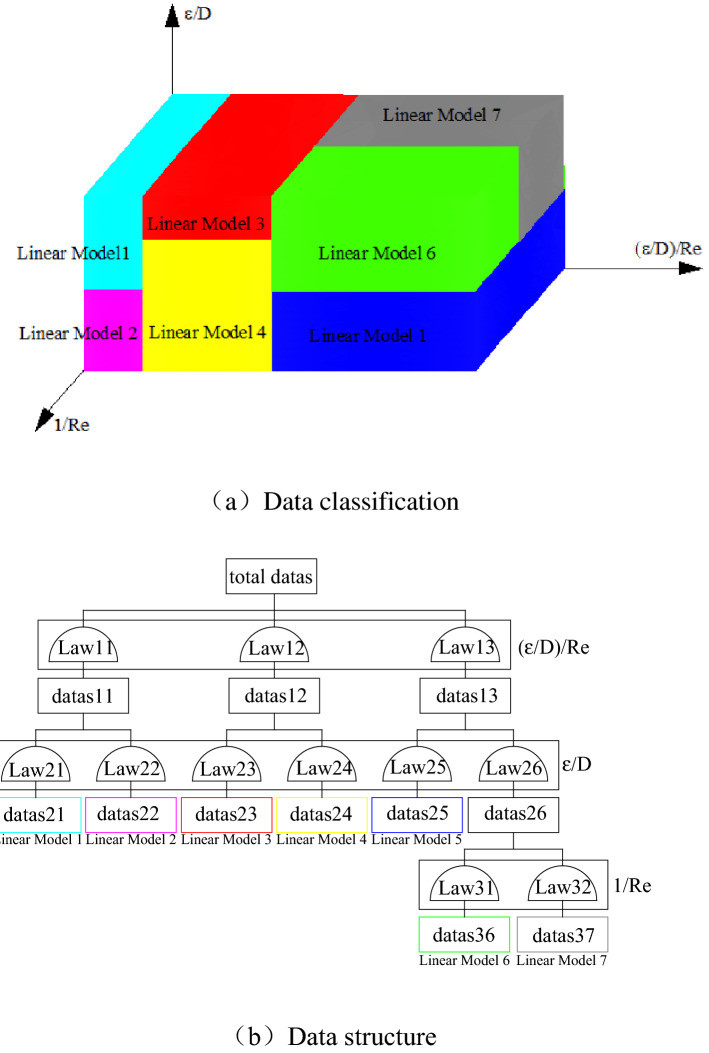
Model tree structure.

Many empirical formulas show that the flow friction factor *f* is related to the relative roughness $\varepsilon /d$ and Reynolds number Re^26–28^. These formulas have errors and have been proven to be effective in the Re range. The constants vary with different roughness prediction algorithms in empirical formulas. The existing empirical formula cannot be satisfied for the fractal roughness algorithm, so a new model of the ventilation resistance coefficient needs to be developed. Because *f*, $\varepsilon /d$ and *Re* are nonlinear combinations, it is necessary to find the relationship among the three parameters with the help of the model tree, which is a nonlinear transform linear processor. The model tree has subjective and spiritual data extraction characteristics as a means of data classification, and it can be used for regression analysis of segmented subsets. Figure 5 shows the sorting of data layer by layer from root nodes to leaf nodes when the model tree processes problems. The tree leaf nodes can represent the problem types and apply a linear multivariate regression model to quantitative analysis of each subdomain. Therefore, the model tree can transform nonlinear approximation into multiple linear model combinations.

Using an iterative method to solve the Colebrook-White equation to develop and test MT. These data are related to laminar, transient, and turbulent states.

13 $$\frac{1}{{\sqrt {\text{f}} }} = - 2{\text{log}}_{10} \left( {\frac{{\varepsilon /{\text{d}}}}{3.7} + \frac{2.523}{{R{\text{e}}\sqrt {\text{f}} }}} \right).$$

According to the relationship between the independent values and dependent variables in the previous empirical formula, the Colebrook–White friction factor is expressed by formula (14). The fractal roughness is introduced into formula (14), and then a new relationship between input and output is established with the developed data sets.

14 $$10^{{\frac{{{ - }0.5}}{{\sqrt {\text{f}} }}}} = {\text{t}}_{0} \cdot \frac{1}{{R{\text{e}}}} + {\text{t}}_{1} \cdot \frac{{\varepsilon_{{\text{f}}} }}{{\text{d}}} + {\text{t}}_{2} \cdot \frac{{\varepsilon_{{\text{f}}} /{\text{d}}}}{{R{\text{e}}}} + {\text{t}}_{3},$$

where, *t*_0_, *t*_1_, *t*_2_, *t*_*3*_ is constant. Thus, the MT method has three inputs and one output parameter.

15 $$f_{{{\text{out}}}} = G_{{{\text{in}}}} \left( {\frac{1}{{R{\text{e}}}},\frac{{\varepsilon_{{\text{f}}} }}{d},\frac{{\varepsilon_{{\text{f}}} /d}}{Re}} \right).$$

A subset contains constants determined by undetermined coefficients. Different subset partition criteria determine different constants. The domain splitting criterion is often used to determine the critical formula segmented subdomains in the model tree^29^. The division rule takes the standard deviation to minimize the expected error reduction at the node. The following formula can express the deviation reduction.

16 $$SDR = {\text{sd}}\left( T \right) - \sum\limits_{{\text{i}}} {\frac{{\left| {T_{{\text{i}}} } \right|}}{\left| T \right|} \times {\text{sd}}\left( {T_{{\text{i}}} } \right)}.$$

*T* is all data sets, and *T*_*i*_ is the data set of each subset node after segmentation. When the class values change very little as they arrive at a node or the data are minimal in the remaining dataset, the segmentation stops.

Further analysis demonstrates that there is a relationship between the resistance factor *f* and ventilation resistance coefficient $\alpha$. The Reynolds number of airflow enters the completely disordered range in most tunnels, and the value is only related to the relative roughness. If the relative roughness is certain, $\lambda$ can be regarded as a constant value for a tunnel with a certain geometric size at standard air density $\rho$ = 1.2 kg/m^3^.

17 $$\alpha = \frac{\lambda \cdot \rho }{8}.$$

$\alpha$ is a function of the relative roughness and air density in complete turbulence. The value of each supporting form is generally obtained by measurement and model tests. The ventilation resistance coefficient of all kinds of tunnels under standard conditions ($\rho$ = 1.2 kg/m^3^) was determined through many experiments. When the air density $\rho_{0}$ ≠ 1.2 kg/m^3^, the value should be corrected as follows:

18 $$\alpha_{0} = \alpha \cdot \frac{\rho }{1.2}.$$

Formula (18) ignores the effect of pressure because the pressure gradient works slightingly. The shear stress of airflow in tunnels is $\tau$. The drag along the tunnel is equal to the pressure of the airflow. The formula (19) is as follows:

19 $$\tau \cdot S{\text{ = p}} \cdot A,$$

where, S is the surface area, m^2^; P is ventilation resistance, Pa; A is the tunnel section area, m^2^. Literature^30^ shows that the shear stress of roughness surface is proportional to the kinetic energy of airflow. The formula (20) is as follows:

20 $$\tau { = }\rho \cdot {\text{f}}\frac{{{\text{v}}^{2} }}{2},$$

where, $\rho$ is the density of airflow, kg/m^3^;v is the velocity of airflow, m/s; *f* is the friction resistance coefficient. Substitute Eq. (20) into Eq. (19) to obtain pressure calculation formula (21).

21 $${\text{p = }}\rho \cdot {\text{f}}\frac{{{\text{S}} \cdot {\text{v}}^{2} }}{2 \cdot A}.$$

Formula (21) indicates the relationship between friction coefficient and pressure. The total surface area, cross-section area, and airflow density of each tunnel are changed slightingly. When there is no ventilation structures interference in the tunnel with a reasonable length, the airflow velocity fluctuates slightly. It can be ignored that the influence of pressure gradient on the prediction of friction coefficient.

## Analysis for an example

### Fractal roughness

**Figure 6:**
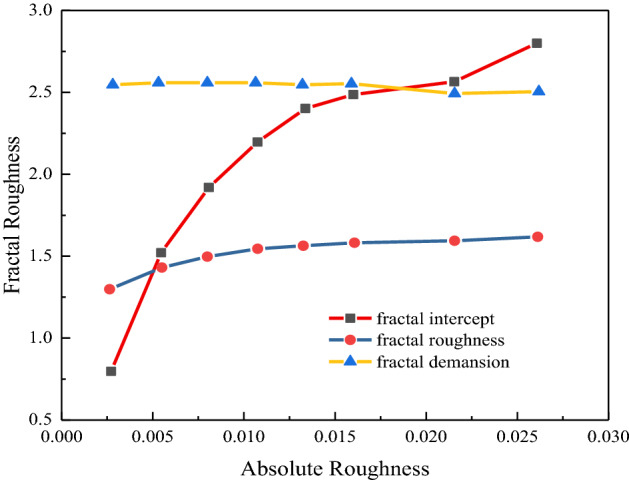
Relationship between absolute roughness and fractal roughness.

The tunnel surface is generated randomly by programming through the point cloud. The tunnel has an arched cross-section (10 m × 4 m × 4 m). The point cloud randomly fluctuates on the smooth roadway surface to represent the random roughness of a surface. Adjacent point clouds are connected to form rough surfaces in the form of triangular meshes. Surfaces with different roughnesses can be obtained by changing the wave coefficient. Fractal calculation results corresponding to the eight absolute roughnesses are expressed in Table 1. The correlation coefficients between the fitting line and scattered points are approximately 0.99, demonstrating that the calculation of fractal parameters is reasonable. The importance is to be the same for coupling the two fractal parameters, so $\omega_{1}$ and $\omega_{2}$ are all valued at 0.5. The investigation of fractal roughness and root mean square roughness demonstrates that the absolute roughness has a slight linear relationship with fractal parameters and fractal roughness. In Fig. 6, it can be seen that the fractal intercept and fractal roughness show an increasing trend with increasing absolute roughness. The fractal dimension remained nearly unchanged and fluctuated slightly between 0.02 and 0.25, reflecting that the fractal intercept can represent the fluctuation of the rough element. The higher rise and fall, the larger the fractal intercept is. The fractal dimension represents the morphological characteristics of the details of roughness elements. Because point clouds have little influence on the morphological details of roughness elements when changing the fluctuation range, the regression trend of the fractal dimension is nearly unchanged. The fractal roughness of the comprehensive fractal dimension and fractal intercept is mainly affected by the fractal intercept. The larger the absolute roughness is, the larger the fluctuation range of the roughness element is. Then the more complex the fractal roughness is.

**Table 1:** Fractal roughness.

No.	Absolute roughness	Fractal dimension	Fractal intercept	correlation factor	$\omega_{1}$	$\omega_{2}$	Fractal roughness
1	0.00268	− 1.1128	0.8115	0.993	0.5	0.5	1.297
2	0.00535	− 1.1162	1.5161	0.993	0.5	0.5	1.427
3	0.00801	− 1.1155	1.9184	0.993	0.5	0.5	1.496
4	0.01067	− 1.1136	2.2016	0.993	0.5	0.5	1.542
5	0.01330	− 1.1042	2.3969	0.993	0.5	0.5	1.573
6	0.01594	− 1.1081	2.4938	0.993	0.5	0.5	1.588
7	0.02154	0.9623	2.5676	0.994	0.5	0.5	1.588
8	0.02618	− 1.0501	2.8013	0.981	0.5	0.5	1.631

### Flow friction factor

The model tree was used to evaluate the friction and flow friction factors in the tunnel. There are $\varepsilon /d$, 1/Re, $\left( {\varepsilon /d} \right)/{\text{Re}}$ three input parameters and *f* one output parameter. In Table 2, $\varepsilon$ ranges from 0.00268 to 0.0261, and Re ranges from 2000 to 10,000. Table 3 presents the nine linear regression tree structures, which split the three input parameters into nine subdatasets. The number of split subsets affects the accuracy of the output result. The split number of the root data set is reduced as much as possible to improve computational efficiency.

**Table 2:** Model tree data set.

Parameters	$\varepsilon$	d	$R{\text{e}}$
Range	0.00268–0.0261	4	2000–10,000

**Table 3:** Model tree parameters.

$\varepsilon /D$	$1/R{\text{e}}$	Fitting lines
0.00067–0.00133	0.0001–0.0003	Line1
	0.0003–0.0005	Line2
0.00133–0.00266	0.0001–0.0005	Line3
0.00266–0.00398	0.0001–0.0003	Line4
	0.0003–0.0005	Line5
0.00398–0.00538	0.0001–0.0002	Line6
	0.0002–0.0005	Line7
0.00538–0.00654	0.0001–0.0004	Line8
	0.0004–0.0005	Line9

The model tree comprises nine lines in the example named from line 1 to line 9, and each linear model maps one equation. t_0_, t_1_, t_2_, and t_3_ were determined by fitting the three input parameters in the sub-data set in Eq. (14). The 9 equations can linearly represent the resistance coefficient ***f*** of the corresponding sub-data set, and the linear model in this region is shown in Table 4.

**Table 4:** Factors in Eq. (14).

Parameters	*t*_0_	*t*_1_	*t*_2_	*t*_4_
Line1	0.00492	10.09425	− 0.54997	− 7.32953 × 10^–4^
Line2	0.00417	8.61436	7.25196	− 8.58096 × 10^–4^
Line3	0.01135	11.09454	− 1.52567	− 0.00335
Line4	0.02873	13.49854	− 6.11323	− 0.01012
Line5	0.02773	10.93078	− 2.7474	− 0.00935
Line6	10.12022	1124.1349	− 2801.38889	− 4.01822
Line7	9.79197	481.68462	− 1187.33945	− 3.88757
Line8	0.02759	12.80927	− 5.39377	− 0.00928
Line9	0.02653	10.66799	− 2.70334	− 0.00843

### Error analysis

**Figure 7:**
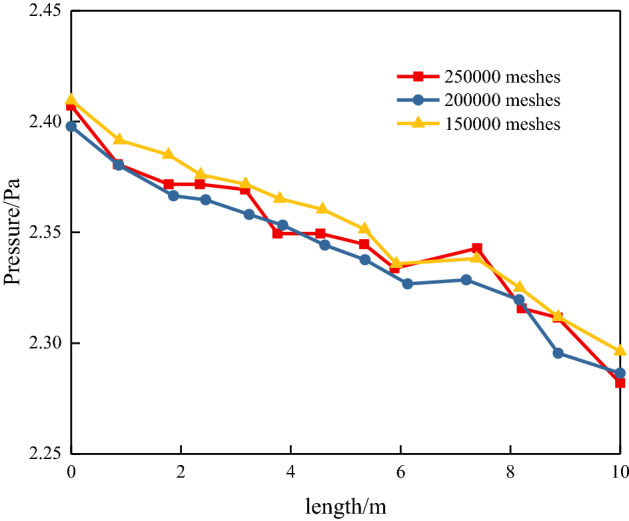
Grid independence verification.

**Figure 8:**
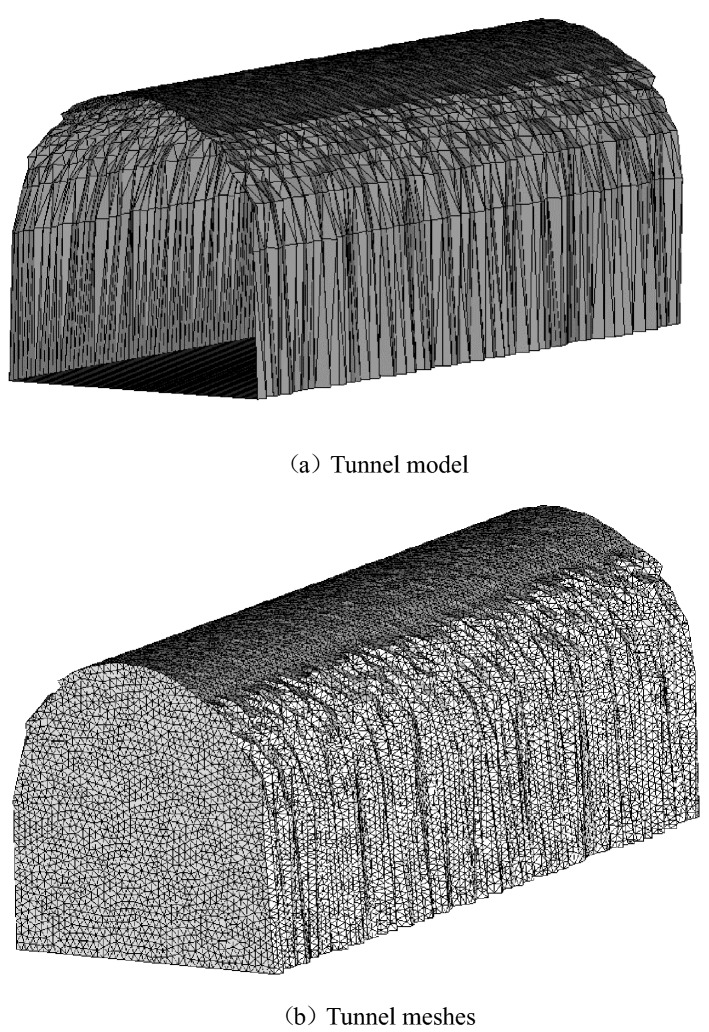
Numerical boundary model.

Figure 7 shows that 150,000 grids has larger deviation than 20,000 and 250,000 grids, indicating that 20,000 grids and 250,000 grids can meet the calculation requirements. As the calculation efficiency of 250,000 grids is lower than 200,000 grids, 20,000 grids are selected as the solution parameter. The simulation results obtained the change law of the pressure drop in the 10 m tunnel. The average pressure of the section under the roughness characteristics of the surrounding rock under the influence of different flow velocities is shown in Fig. 8. A comparison between the Colebrook calculation and model is shown in Table 5. The error is between 0.7 and 8%, in which the error of Line 4 is the smallest and that of Line 3 and Line 9 is the largest.

**Table 5:** Flow resistance factor prediction and numerical simulation prediction.

Type	Line1	Line2	Line3	Line4	Line5	Line6	Line7	Line8	Line9
α × 10^–3^	6.272	6.114	6.103	6.098	8.114	4.711	7.114	6.114	6.361
α × 10^–3^_(Colebrook)_	6.215	6.543	6.606	6.146	7.873	4.996	7.233	6.543	7.870
$\delta$	0.9%	7%	8%	0.7%	2.9%	6.1%	1.6%	7.0%	8.0%

The rule of pressure drop under different flow velocities was obtained by numerical simulation. The sections of the central axis of the tunnel strike as the verification example, which consists of 4400 points and 2,700,000 meshes, are shown in Fig. 8. The model has a 14.28 m^2^ arch cross section 10 m in length. In the numerical simulation, we choose the *k*-ω (standard) mathematical turbulence model with the solver based on pressure. The model achieves more accurate near-wall processing by automatically switching from the wall function to the Reynolds number formula based on mesh spacing. The superior performance of the wall boundary and low Reynolds number flow is demonstrated. The fluid inside the boundary is air with a density of 1.29 kg/m^3^, and the viscosity coefficient is 1.8 × 10^–5^ Pa/s. One side of the model is the air inlet, where the velocity inlet is set as 0.25 m/s, 1 m/s, 1.5 m/s, and 2 m/s. The other should be Outflow freely; All residual tests are set as 1 × 10^–5^. The convergence calculation ends at approximately 1300 steps. The coupling algorithm is adopted because the airflow in the calculation region is affected by the rough wall surface and is turbulent. Pressure drop law conforms to formula (22):

22 $$H = \left| {P_{1} - P_{2} } \right| = \frac{\alpha ^{\prime} \cdot L \cdot U}{{S^{3} }}Q^{2}$$

where *H* is the pressure difference between two sections; *P*_1_ is the midpoint pressure at the inlet section, *P*_2_ is the midpoint pressure at the outlet section, $\alpha ^{\prime}$ is the flow friction factor, *L* is expressed as the tunnel length; *U* is the wet circumference, *S* is the sectional area, and *Q* is the flow velocity in the middle of the section. The simulation results obtain the average pressure of the tunnel section. Then, the flow friction factor has been calculated in reverse. The relative error between the simulation result and the model result can be expressed by formula (23):

23 $$\delta = \frac{{\left| {\alpha - \alpha ^{\prime}} \right|}}{\alpha }$$

**Figure 9:**
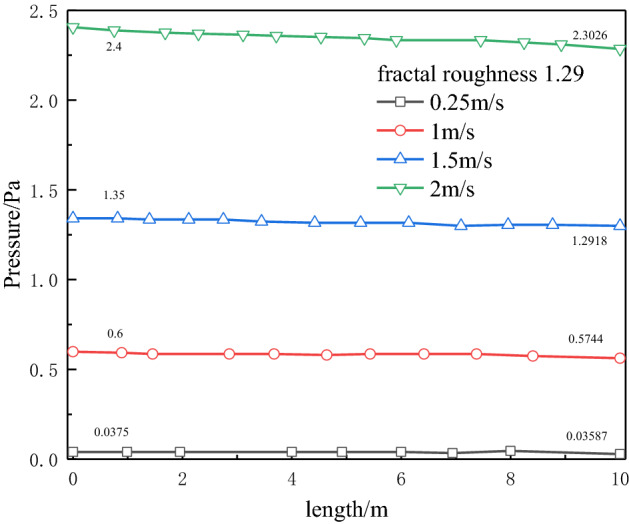
Average pressure drop in roadway section.

The wind speeds are 0.25 m/s, 1 m/s, 1.5 m/s and 2 m/s when the fractal roughness is 1.29. While the roughness is constant, the pressure increases with increasing flow velocity in the tunnel, as shown in Fig. 9. According to formulas (19) and (20), the ventilation resistance coefficient is unchanged, and the calculated results are shown in Table 6.

**Table 6:** Friction factor at different flow velocities.

Velocity/ m/s	P_1_/Pa	P_2_/Pa	$\alpha$	$\alpha ^{\prime}$	$\delta$ (%)
0.25	0.0375	0.03587	0.00259	0.00253	2.31
1	0.600	0.5744	0.00254	0.00253	2.36
1.5	1.35	1.2918	0.00256	0.00253	1.21
2	2.412	2.3026	0.00250	0.00253	1.18

Table 6 shows the simulation results and theoretical results. The inlet pressures are 0.0375 Pa, 0.600 Pa, 1.35 Pa, and 2.412 Pa. The simulated outlet pressures are 0.03587 Pa, 0.5744 Pa, 1.2918 Pa, and 2.3026 Pa. The friction resistance coefficients of ventilation are calculated to be 0.00259, 0.00254, 0.00256, and 0.00250 according to formula (22) and roadway size. Compared with the model in this paper, the relative error is the largest when the wind speed is 1 m/s, and the smallest when the wind speed is 2 m/s, indicating that the ventilation resistance coefficient has nothing to do with the air velocity. The fluctuation of small error indicates that the model can accurately calculate the low friction coefficient.

## Conclusions

The influence of pressure and temperature is ignored in this study. In addition, the density change in the tunnel is regarded as a constant value. The ventilation resistance coefficient is regarded as the inherent attribute to the tunnel, and the calculation model is based on the surface point cloud as the surrounding rock distribution. The following conclusions are obtained:

1. The combination results of fractal dimension and fractal intercept are taken as roughness parameters by fractal theory. We also carried out a calculation method of friction drag based on fractal characterization. It can be better characterized that the influence of the concave and convex characteristics of three-dimensional rough surfaces on friction wind resistance compared with the relative roughness parameters in the empirical formula.
2. The three-dimensional laser scanner was programmed to replace the tunnel point cloud data. The airflow in the tunnel was regarded as complete turbulence. The friction coefficient error between the new method and the numerical simulation is less than 3%. The calculation method of friction factor based on fractal theory has high accuracy. Use three-dimensional laser scanning technology to obtain the friction resistance factor support for the realization of intelligent ventilation.
3. Pressure and temperature affect the flow, but we ignore changes in temperature and pressure. On the other hand, the dimension weights and intercept weights are transformed directly and added to obtain the fractal roughness in this paper.
